# In Vitro Biomechanical Experiment on the Effect of Unilateral Partial Facetectomy Performed by Percutaneous Endoscopy on the Stability of Lumbar Spine

**DOI:** 10.3390/bioengineering12040414

**Published:** 2025-04-14

**Authors:** Tao Ma, Xiaoshuang Tu, Junyang Li, Jingwei Wu, Luming Nong

**Affiliations:** 1 Orthopedics Department, The Third Affiliated Hospital of Nanjing Medical University, Changzhou 213164, China; matao@stu.njmu.edu.cn (T.M.); 13330732948@163.com (X.T.); 2024122109@stu.njmu.edu.cn (J.L.); wujingwei1999@outlook.com (J.W.); 2 Orthopaedics Department, Nanjing Medical University, Nanjing 211103, China; 3 Changzhou Medical Center, Nanjing Medical University, Changzhou 213164, China; 4 The Affiliated Changzhou No.2 People’s Hospital of Nanjing Medical University, No.29 Xinglong Lane, Tianning District, Changzhou 213000, China

**Keywords:** biomechanics, bioengineering, preclinical studies, therapeutics, unilateral partial facetectomy

## Abstract

Objectives: This study’s purpose is to investigate the lumbar biomechanical effects of unilateral partial facetectomy (UPF) of different facet joint (FJ) portions under percutaneous endoscopy. Methods: Forty fresh calf spine models were used to simulate UPF under a physiological load performed through three commonly used needle insertion points (IPs): (1) The apex of the superior FJ (as the first IP); (2) The midpoint of the ventral side of the superior FJ (as the second IP); (3) The lowest point of the ventral side of the superior FJ (as the third IP). The range of motion (ROM) and the L4/5 intradiscal maximum pressure (IMP) were measured and analyzed under a physiological load in all models during flexion, extension, left–right lateral flexion, and left–right axial rotation. Results: When UPF was performed through the second IP, the ROM of the lumbar spine and the L4/5 IMP in the calf spine models were not statistically different from the intact calf spine model. Conclusions: UPF through the second IP resulted in a minimal impact on the biomechanics of the lumbar spine. Thus, it might be considered the most appropriate IP for UPF.

## 1. Introduction

Lumbar hypermobility following traditional open spinal surgery is a major cause of chronic low back pain, affecting long-term patient outcomes [1,2]. This instability often results from the disruption of spinal anatomical structures during surgical interventions. To address these limitations, percutaneous endoscopic transforaminal discectomy (PETD) has emerged as a minimally invasive alternative, offering reduced tissue damage and faster recovery [3,4]. However, a critical challenge in PETD is the unavoidable partial resection of the facet joint (FJ) to access the spinal canal [5,6,7,8]. Clinical studies indicate that such resection may lead to postoperative hypermobility [9,10], underscoring the need to balance decompression with biomechanical preservation. The facet joints are pivotal in maintaining spinal stability by resisting compression, shear, and rotational forces [11,12]. Disruption of their integrity redistributes stress to adjacent structures, accelerating degeneration [13,14]. Prior biomechanical studies primarily focused on facetectomy volume [15], yet neglected the impact of resection location—a critical factor in clinical practice where surgeons select specific insertion points (IPs). This study aims to systematically evaluate the biomechanical effects of unilateral partial facetectomy (UPF) performed through three clinically relevant IPs. By measuring intradiscal pressure (IMP) and range of motion (ROM), we identify the optimal IP that minimizes biomechanical disruption.

## 2. Materials and Methods

### 2.1. Calf Spine Specimen Preparation

Fifty fresh calf lumbar segments (L3–L6) were collected from 15-month-old calves with uniform weight and spinal status, and all specimens were X-rulated for structural integrity, excluding fractures, tumors, and severe osteoporosis, and 40 were used in follow-up experiments. All specimens were wrapped in gauze soaked in saline, sealed in a double-layer plastic bag, and stored at −20 °C. Before testing, specimens were first thawed at 4 °C for 12 h to minimize structural damage [16], followed by an additional 8 h thawing period at room temperature. During this period, ice cubes were placed around the specimens to maintain a low temperature and prevent tissue degeneration. Subsequently, muscle and adipose tissue were carefully removed, preserving the integrity of the bony structures, intervertebral discs, facet joints, and ligaments. The integrity of the bony structure, lumbar intervertebral disc, FJ, and ligament tissue should be maintained by paying attention during the handling of the specimen, which was then covered with gauze soaked in saline to keep it moist. Since the calf specimens possessed a long transverse process, all calf specimens were partially cut (retaining approximately 7 cm in length) in order to facilitate the experimental operation. The two ends of the L3 and L6 segments of the specimen were fixed in a special test mold using Kirschner wires, embedded in self-curing denture powder (dental polymethyl methacrylate), and kept horizontal to facilitate the installation of the specimen on the loading device.

### 2.2. Insertion Point (IP) Determination

According to the actual clinical surgery method, facetectomy was performed by removing the ventral side of the superior FJ up to the dorsal side. Thus, three commonly used clinical needle IPs were selected: (1) The apex of the superior FJ (as the first IP); (2) The midpoint of the ventral side of the superior FJ (as the second IP); (3) The lowest point of the ventral side of the superior FJ (as the third IP). In this experiment, a trephine with a diameter of 7.5 mm commonly used in clinical surgery was used to simulate the UPF.

### 2.3. Facetectomy Simulation and Grouping

#### Calf Spine Model

**Figure 1:**
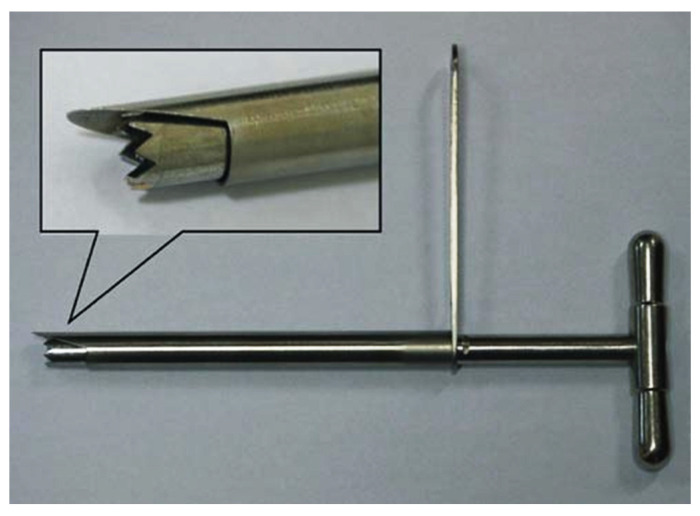
The trephine used in the biomechanical experiment of calf spine specimens.

**Figure 2:**
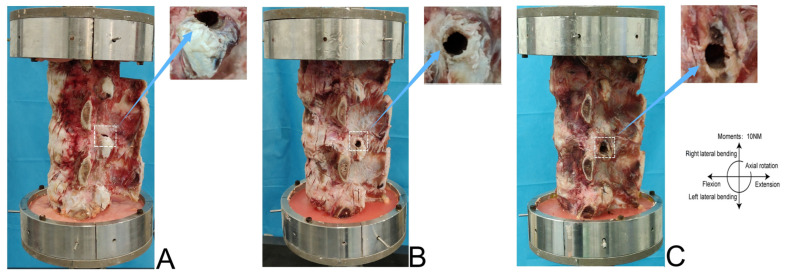
The schematic diagram of L5 left superior facet joint resection in the calf spine specimens: (**A**) Group A. (**B**) Group B. (**C**) Group C.

Figure 1 shows the percutaneous lumbar facetectomy instrument used in the experiment. First, the tip of the duckbill protective sleeve was pushed from the lateral posterior approach through the lower half of the left intervertebral foramen of the specimen to the level of the upper endplate of the L5. The oblique opening of the sleeve faced the back side of the lumbar spine and the sleeve pressed against the IP on the L5 superior FJ. The sleeve formed an angle of 20° with the coronal surface of the lumbar spine specimen and was parallel to the plane of the intervertebral disc. The intervertebral foramen was formed and enlarged by the matching trephine through the inner cavity of the protective sleeve. The inner diameter of the enlarged protective sleeve was 8.0 mm, and the outer diameter of the trephine was 7.5 mm. Forty fresh calf spine specimens were randomly divided into 4 groups with 10 specimens in each group. The control group was represented by the intact calf spine. Group A was represented by the calf spine model in which UPF was performed through the first IP. Group B was represented by the calf spine model in which UPF was performed through the second IP. Group C was represented by the calf spine model in which UPF was performed through the third IP. Figure 2 shows the calf spine models after UPF.

### 2.4. Model Boundaries and Load Conditions

#### Calf Spine Model

**Figure 3:**
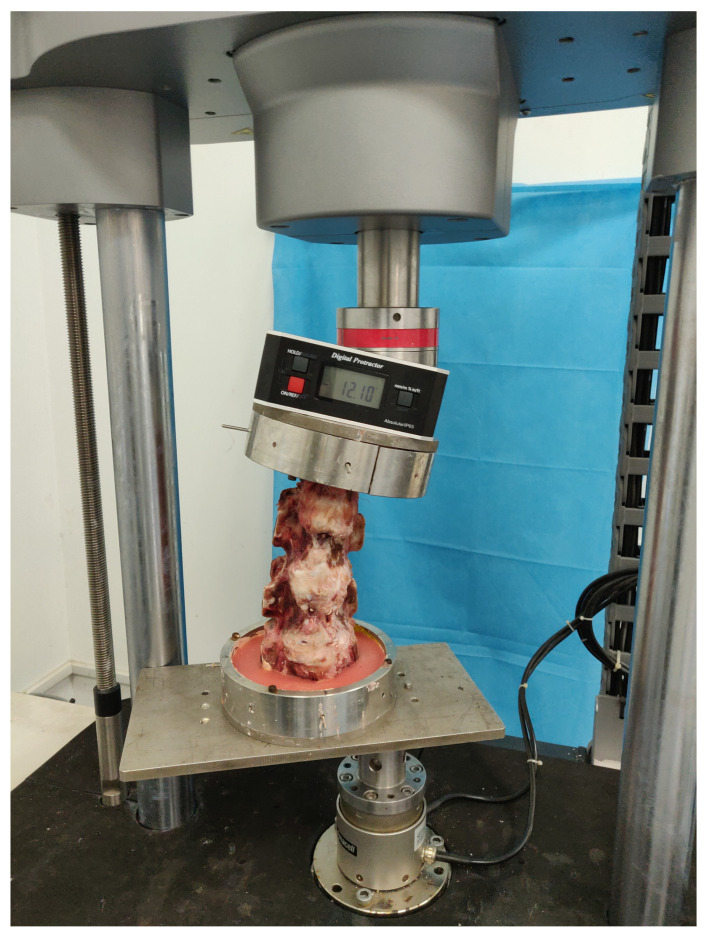
The Intron E10000 tension and torsion biaxial universal material biomechanical testing machine and electronic digital level for calf spine specimens biomechanical test.

IMP and ROM were chosen as the core parameters because they reflect intervertebral disc load distribution (IMP) and spinal range of motion (ROM), respectively, which are the gold standards for evaluating postoperative stability. A scalpel was used to perform a horizontal incision of 1 cm parallel to the endplate of the vertebral body in the center of the L4/5 intervertebral disc, the needle to test the pressure (approximately 1 mm in diameter) was inserted parallel to the endplate of the vertebral body into the posterior edge of the intervertebral disc, and the Gaeltec pressure sensor was connected to measure the IMP. Before the test, the calf spine specimens were fixed on the Intron E10000 tension and torsion biaxial universal material biomechanical testing machine using a special fixture for testing calf spine mechanics. An electronic digital level for a spine biomechanical test installed above the special fixture was present to measure lumbar spine ROM (Figure 3). According to the standards of the calf spine specimen test proposed by Wilke et al. [17], the torque was set to 10 N·m and the constant axial force was set to 400 N. The specimens were loaded and unloaded twice to remove their viscoelasticity before measuring the ROM of the calf spine flexion, extension, lateral bending, axial rotation, and the L4/5 IMP, in order to ensure the accuracy of the data, and the results were recorded on the third round of measurements, so that relatively stable kinematic test data could be obtained. The specimens were continuously sprayed with saline during the entire test to keep them moist and minimize tissue degeneration.

### 2.5. Statistical Analysis

Statistical analysis was performed using SPSS (version 22.0; IBM Corp., Armonk, NY, USA). The mean and standard deviation (SD) of IMP and ROM were calculated using one-way ANOVA and the measured data were expressed as “x ± s”. All reported *p* values were two-tailed, and *p* values less than 0.05 were considered statistically significant.

## 3. Results

### 3.1. Calf Spine Models

#### 3.1.1. L4/5 IMP

The data of the pressure sensor in the L4/5 intervertebral disc of the calf spine specimen were recorded and analyzed. The L4/5 IMP showed a significant increase among Groups A, B, C, and the control group in flexion, extension, left–right lateral flexion, and left–right axial rotation. The L4/5 IMP significantly increased under extension in Group A compared with the control group (*p* < 0.05). The L4/5 IMP slightly increased in Group B compared with its value in the control group during extension and left–right axial rotation, but the difference was not statistically significant. The difference in flexion and left–right lateral flexion was also not statistically significant compared with the control group. Compared to the control group, in flexion, extension, left–right lateral flexion, and left–right axial rotation, the L4/5 IMP (intervertebral disc space) in Group C showed a significant increase. (*p* < 0.05). The detailed data are listed in Table 1.

#### 3.1.2. ROM

The ROM of the calf spine specimens was obtained by recording the electronic digital level on the upper end of the specimen fixture (Table 2). There was a significant increase in ROM between Group A, B, C and the control group in left–right axial rotation, with no significant differences in flexion, extension, and left–right lateral flexion. No significant difference in ROM was found under the six motions of flexion and extension, left–right lateral flexion, and left–right axial rotation in Group A and Group B compared with the control group. No significant difference in ROM was found under the four motions of flexion and extension, left–right lateral flexion and left–right axial rotation in Group C compared with the control group. However, the ROM was significantly increased under left–right axial rotation in Group C compared with its value in the control group (*p* < 0.05).

## 4. Discussion

This study evaluated the biomechanical effects of three facet excision approach points using a bovine lumbar spine model. The results showed that the second approach (ventral midpoint) performed best in preserving spinal stability, while the first and third approaches resulted in increased disc pressure and abnormal range of motion, respectively. These findings provide a key biomechanical basis for optimizing percutaneous endoscopic surgery (PETD).

Our findings demonstrate that the choice of insertion point (IP) during unilateral partial facetectomy (UPF) critically impacts lumbar spine stability [18,19,20,21]. While previous studies focused on facetectomy volume thresholds, our work uniquely evaluates the biomechanical effects of UPF performed through different IPs, aligning with clinical surgical practices [1,22,23]. At present, lumbar hypermobility is diagnosed by the relative displacement or angle of the vertebral body in the flexion and extension positions. Hasegawa K. et al. also believed that the increase in the FJ space is the strongest predictor of lumbar hypermobility [24]. Therefore, the integrity and health of the FJs are essential in the stability of the lumbar spine. The best choice for testing the biomechanics of lumbar spine is the use of specimens from a fresh human cadaver. However, it is difficult to obtain a sufficient number of cadavers of the same age and gender. Calf vertebral bodies are similar in size to the human ones and have a wide variety of sources. Therefore, the experiments in this work were performed using 40 calf spine specimens to perform UPF.

During the PETD surgery, an insertion operation is performed under local anesthesia in the posterior aspect of the vertebral body to allow direct entry into the spinal canal to perform the discectomy. The surgery is performed far from the outlet and dorsal root ganglia, avoiding as much as possible the muscles and ligaments adjacent to the vertebral body, but a partial destruction of the FJ is inevitable. Some biomechanical studies showed that the destruction of FJs increases the risk of spinal hypermobility and spinal degeneration [25,26,27].

However, recent advances in finite element (FE) research on facetectomy have been made. In 2014, Erbulut [28] performed an FE analysis and found that the FE model of the lumbar spine is severely affected in extension and axial rotation after the complete removal of one FJ side. Thus, lumbar fusion or pedicle screw fixation is required after the removal of the bilateral FJs of the lumbar spine. However, in 2017, Zeng et al. [7] simulated the resection of the 50% of one FJ side on a lumbar FE model and they realized that the intradiscal pressure and intervertebral ROM were not significantly different from their value in the intact model. Thus, these patients do not need lumbar fusion or lumbar fixation. On the other hand, in 2019, Li et al. [29] simulated the graded resection of the lumbar FJs through FE analysis and discovered that the removal of 50% of the unilateral lumbar FJ increased the risk of biomechanical degeneration of the lumbar spine and the occurrence of failed back surgery syndrome. In 2020, S. Ahuja et al. [15] performed an FE analysis and discovered that the lumbar spine ROM, the pressure in the FJs, and the pressure in the intervertebral disc significantly increase when more than 30% of a unilateral facet joint is removed. The conclusions of the above studies on the biomechanics of facetectomy are different, because none of these studies established a unified standard in the resection of the lumbar FJs. The experiments performed in previous works were mostly resection of the superior FJ from the dorsal side to the ventral side, which is not in agreement with what is performed in clinical practice, and the cutting method was a longitudinal cut of the artificial division [8,15,28,29,30,31]. Indeed, a trephine is used in clinical practice to perform a cut from the ventral side of the superior FJ to the dorsal side, and from the head to the tail end [32,33,34]. The resected superior FJ is left with an arc-shaped gap. Therefore, the previous studies are not applicable in clinical surgery, thus having less significance.

The three IPs selected in our experiments were needle IPs currently used in clinical surgery, with the first IP (the apex of the superior FJ) as the most commonly used IP. The percutaneous endoscopic discectomy was invented in 1993 by the German spine surgeon Hoogland Thomas [35] who chose the apex of the superior FJ as the needle IP [4,36]. Therefore, this IP was selected also in this work as one of the IPs to perform the experiments. In our experiment, the height of the L5 superior FJ of the lumbar FE model was 13.4 mm, the average height of the L5 superior FJ of the calf spine models was greater than 13 mm, and the radius of the trephine was 3.75 mm, thus, the height of the superior FJ was sufficient to divide the three IPs, all placed at the edge of the superior FJ. Facetectomy performed through the ventral edge of the superior FJ can maximize the expansion of the intervertebral foramen. In addition, the test data on these three IPs could be compared with each other to evaluate the impact of facetectomy of the different FJ portions on the biomechanics of the lumbar spine.

The bilateral FJs and the intervertebral disc form a spine unit that shares the pressure of the trunk on the vertebral body [37,38,39]. Since the top of the superior FJ is triangular, a stress concentration may easily occur at the tip of the superior FJ under physiological conditions, meaning that the stress sustained in this point is relatively large. The facetectomy performed through the first IP resulted in the destruction of the integrity of the superior FJ, leading to stress redistribution, and the larger stress sustained by the apex of the superior FJ was distributed to the adjacent intervertebral disc and the contralateral FJ. The first IP (the apex of the superior FJ) was the most commonly used IP. The in vitro biomechanics test revealed no significant difference in the ROM of the lumbar spine after facetectomy of the calf spine models between Group A compared with the control group. However, compared with the control group, the L4/5 IMP under extension in Group A was significantly increased. This might be related to the protection of the joint capsule of the FJs. Indeed, some studies showed that the joint capsule of the FJs not only protects and nourishes the FJs, but also shares the physiological load, thus limiting the excessive movement of the lumbar spine [9,40,41]. The second IP was located on the outer edge of the intervertebral foramen. In the in vitro biomechanics test, no statistically significant difference was found in the L4/5 IMP and ROM of the lumbar spine in Group B compared with the control group. The third IP was performed in the thickest part of the superior FJ, where the stress was the largest. The in vitro biomechanics test revealed a statistically significant difference in the L4/5 IMP and ROM of the lumbar spine in Group C compared with the control group.

This work has a limitation. No specimens from cadavers were used to perform the in vitro biomechanical studies, thus, calf spine specimens were preferred since calf vertebral bodies are similar in size to the human ones, and because calf spine specimens represent the best replacement for cadaver spine specimens to perform biomechanical research. However, in the present experiments, the FJs of the calf spine specimens possessed thicker joint capsules and greater bone density, which increased the difficulty in the removal of the excess muscle and soft tissues from the specimens. The outer diameter of the trephine used in the experiment was 7.5 mm, therefore, the biomechanical effect of other size trephine or tools used for facetectomy on the lumbar spine needs further study and additional clinical research.

## 5. Conclusions

This study systematically evaluated the biomechanical effects of unilateral partial facetectomy (UPF) performed through three insertion points (IPs) in calf spine models. Key findings include: Firstly, UPF through the second IP (midpoint of the ventral superior facet joint) minimized biomechanical disruption, with no significant differences in ROM or L4/5 IMP compared to intact specimens. Secondly, UPF through the first IP (apex of the superior facet joint) increased L4/5 IMP during extension, suggesting potential risks for postoperative instability. Finally, the third IP (lowest ventral point) induced significant biomechanical degradation, validating its unsuitability for clinical application. These results emphasize the importance of IP selection in UPF procedures. UPF performed through the midpoint of the ventral side of the superior FJ resulted in a minimal impact on the biomechanics of the lumbar spine, suggesting that it might be the most appropriate IP for UPF.

## Figures and Tables

**Table 1:** The L4/5 intradiscal maximum pressure of calf spine specimens after facetectomy (Kpa).

Grouping	Control	A	B	C	*F* Value	*p* Value
Flexion	572.48 ± 23.38	694.91 ± 29.65	654.64 ± 15.99	729.48 ± 24.22 ^a^	54.926	*p* < 0.001
Extension	627.53 ± 41.22	941.51 ± 39.62 ^a^	714.05 ± 41.35	1000.23 ± 52.32 ^a^	66.921	*p* < 0.001
Left lateral flexion	556.37 ± 43.42	682.89 ± 51.74	638.08 ± 33.68	715.79 ± 46.29 ^a^	78.891	*p* < 0.001
Right lateral flexion	578.47 ± 52.12	705.20 ± 55.48	664.88 ± 43.15	738.40 ± 46.53 ^a^	68.212	*p* < 0.001
Left axial rotation	685.28 ± 37.42	794.08 ± 48.26	742.27 ± 33.82	1123.47 ± 33.58 ^a^	88.651	*p* < 0.001
Right axial rotation	663.78 ± 46.96	786.45 ± 45.92	725.11 ± 47.15	1047.60 ± 51.62 ^a^	85.621	*p* < 0.001

Note: Compared to the control group, ^a^ *p* < 0.05.

**Table 2:** The range of motion of calf spine specimens after facetectomy (°).

Grouping	Control	A	B	C	*F* Value	*p* Value
Flexion	10.23 ± 2.09	11.44 ± 3.29	11.55 ± 2.12	12.68 ± 3.11	2.052	*p* > 0.05
Extension	7.20 ± 1.77	7.85 ± 1.64	7.65 ± 0.84	8.15 ± 1.14	1.986	*p* > 0.05
Left lateral flexion	8.33 ± 1.27	8.95 ± 1.19	8.90 ± 1.11	10.45 ± 1.53	1.999	*p* > 0.05
Right lateral flexion	8.85 ± 1.25	9.47 ± 1.34	8.95 ± 1.18	10.15 ± 1.20	1.879	*p* > 0.05
Left axial rotation	4.80 ± 0.75	5.35 ± 0.68	5.20 ± 0.76	9.23 ± 1.50 ^a^	10.598	*p* < 0.001
Right axial rotation	5.85 ± 0.84	6.35 ± 0.67	6.55 ± 0.75	10.15 ± 1.44 ^a^	12.368	*p* < 0.001

Note: Compared to the control group, ^a^ *p* < 0.05.

## Data Availability

The data that support the findings of this study are available from the corresponding author upon reasonable request.
